# Establishment of an RNA polymerase II-driven reverse genetics system for Nipah virus strains from Malaysia and Bangladesh

**DOI:** 10.1038/s41598-019-47549-y

**Published:** 2019-08-01

**Authors:** Bryan D. Griffin, Anders Leung, Mable Chan, Bryce M. Warner, Charlene Ranadheera, Kevin Tierney, Jonathan Audet, Kathy L. Frost, David Safronetz, Carissa Embury-Hyatt, Stephanie A. Booth, Darwyn Kobasa

**Affiliations:** 1 Special Pathogens Program, National Microbiology Laboratory, Public Health Agency of Canada, 1015 Arlington Street, Winnipeg, Manitoba R3E 3R2 Canada; 2 Department of Medical Microbiology and Infectious Diseases, University of Manitoba, 745 Bannatyne Avenue, Winnipeg, Manitoba R3E 0J9 Canada; 3 Molecular Pathobiology, National Microbiology Laboratory, Public Health Agency of Canada, 1015 Arlington Street, Winnipeg, R3E 3R2 Manitoba Canada; 4 National Centre for Foreign Animal Disease, Canadian Food Inspection Agency, 1015 Arlington Street, Winnipeg, Manitoba R3E 3M4 Canada

**Keywords:** Viral infection, Viral pathogenesis

## Abstract

Nipah virus (NiV) has emerged as a highly lethal zoonotic paramyxovirus that is capable of causing a febrile encephalitis and/or respiratory disease in humans for which no vaccines or licensed treatments are currently available. There are two genetically and geographically distinct lineages of NiV: NiV-Malaysia (NiV-M), the strain that caused the initial outbreak in Malaysia, and NiV-Bangladesh (NiV-B), the strain that has been implicated in subsequent outbreaks in India and Bangladesh. NiV-B appears to be both more lethal and have a greater propensity for person-to-person transmission than NiV-M. Here we describe the generation and characterization of stable RNA polymerase II-driven infectious cDNA clones of NiV-M and NiV-B. *In vitro*, reverse genetics-derived NiV-M and NiV-B were indistinguishable from a wildtype isolate of NiV-M, and both viruses were pathogenic in the Syrian hamster model of NiV infection. We also describe recombinant NiV-M and NiV-B with enhanced green fluorescent protein (EGFP) inserted between the G and L genes that enable rapid and sensitive detection of NiV infection *in vitro*. This panel of molecular clones will enable studies to investigate the virologic determinants of henipavirus pathogenesis, including the pathogenic differences between NiV-M and NiV-B, and the high-throughput screening of candidate therapeutics.

## Introduction

Nipah virus (NiV), a member of the family *Paramyxoviridae* and genus *Henipavirus* along with the closely related Hendra virus (HeV), is a recently emergent cause of severe morbidity and mortality in humans, with clinical manifestations that can include an often fatal, acute febrile encephalitis and/or pulmonary syndrome^1,2^. NiV has been shown to infect a broad range of vertebrate species^3–7^, including fruit bats belonging to the genus *Pteropus*, commonly known as flying foxes, which have been recognized as the primary natural NiV reservoir^8,9^. There are two distinct NiV genotypes, NiV-M (Malaysia) and NiV-B (Bangladesh), that have been responsible for past outbreaks in humans following a zoonotic spillover event^10,11^. The initial documented outbreak of NiV-M occurred in Malaysia in 1998 when an outbreak of febrile encephalitis was observed among pig farmers, resulting in over 280 people infected with an approximate 40% case fatality rate (CFR) with a very low incidence of human-to-human transmission^12–18^. In this outbreak, pigs were recognized to be an intermediate, amplifying host of NiV, and the government mandated the culling of approximately 1.1 million pigs to halt the outbreak^18^. Since the year 2000, NiV has been detected in flying foxes in Malaysia, but no cases of human infection have been reported^19^. In contrast, smaller NiV-B outbreaks have occurred periodically in India^20–23^ and on an almost yearly basis in Bangladesh^10,24,25^. In contrast to the initial outbreak in Malaysia, outbreaks in Bangladesh have had a markedly higher CFR of approximately 67–92%^25–27^, have not been associated with amplification in pigs but rather with the consumption of fruit or raw date palm sap contaminated with bat urine or saliva^27,28^, and person-to-person transmission has been observed^26,27^.

While a HeV glycoprotein subunit vaccine (sGHeV) has been licensed in Australia to protect against HeV infection in horses^29^ and both sGHeV and a post-exposure neutralizing antibody treatment (m102.4) have demonstrated pre-clinical efficacy against NiV infection in African green monkeys^30,31^ there are currently no approved vaccines or therapeutics against NiV. Further, the effectiveness of treatment with antivirals, such as ribavirin, against NiV has not been definitively demonstrated *in vivo*^32,33^. NiV and HeV have been identified as agents of biodefense concern^34^ with pandemic potential^35^. By virtue of the high case fatality rate and lack of approved or licensed vaccines and therapeutics, work with NiV must be performed under biosafety level 4 (BSL4) conditions.

NiV has a single-stranded, negative-sense, nonsegmented RNA genome that can range in size from 18,246 nucleotide (nt) (NiV-M) to 18,252 nt (NiV-B) in length^36^. The genomes of NiV-M and NiV-B have the same organization, encoding a nucleoprotein (N), phosphoprotein (P), matrix protein (M), fusion protein (F), glycoprotein (G), and large protein (L)^37^; and despite the pathogenic differences between NiV-M and NiV-B^38^, the two genotypes share an overall nucleotide homology of 91.8%^11^. Reverse genetics systems have been developed for HeV^39,42^, Cedar virus, a non-pathogenic henipavirus^40^, and NiV-M^41–43^, which has enabled insightful functional studies of henipavirus pathogenesis^42–46^; however, despite NiV-B being the agent responsible for every outbreak since 1998/99, with the possible exception of an outbreak in the Philippines in 2014^23^, a reverse genetics system for NiV-B has yet to be reported. The development of recombinant NiV-M encoding a marker gene such as EGFP or luciferase has enabled high-throughput drug screening^47,48^. Strategies to develop recombinant henipaviruses have evolved over the last 10 years: the initial recombinant henipavirus (NiV-M) expressing a foreign reporter gene was generated with the marker gene inserted between the N and P genes^41^; however since foreign gene insertion can interfere with the polar transcription gradient of paramyxoviruses^49^ subsequent recombinant henipaviruses have been constructed with the marker gene placed between the P and M genes^39,40^ or at the 5′ end of the M gene to generate a self-cleaved fusion protein^42,47^. Further, the initial recombinant NiV depended on recombinant vaccinia virus that expressed the bacteriophage T7 polymerase (T7pol) for rescue^41^, whereas successive recombinant henipaviruses have employed co-transfection with a T7pol expression construct^39,42^ or rescue in a cell line that stably expresses T7pol^43^. Additional technical refinements have improved rescue efficiency such as optimized self-cleaving hammerhead ribozyme sequences^42^ and optimized P2A ribosomal skipping/cleavage sequence^50^, required to process transcribed RNA to match vRNA sequences.

Here we report a highly efficient DNA dependent RNA polymerase II (pol II)-driven reverse genetics system for both NiV-M and NiV-B that does not rely on T7pol expression and produces virus that replicates with wild-type kinetics and recapitulates the described disease in Syrian hamsters. We also report a recombinant NiV-M and NiV-B, NiV-M/G-EGFP and NiV-B/G-EGFP, respectively, that express EGFP from an open reading frame (ORF) inserted between the G and L genes, enabling rapid and sensitive detection of NiV infection and the determination of NiV titers *in vitro*. We further demonstrate in a proof-of-concept study that NiV-M/G-EGFP and NiV-B/G-EGFP support rapid, high-throughput screening for therapeutic antibodies of interest to protect against NiV infection.

## Results

### Design of rgNiV-M and rgNiV-B viruses

**Figure 1:**
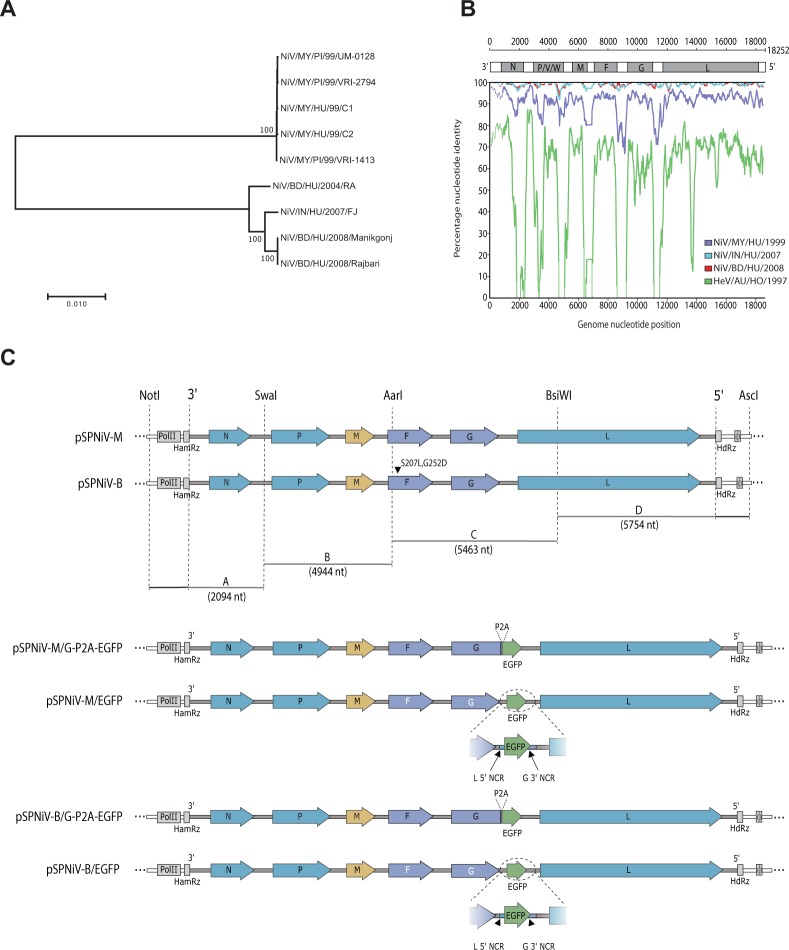
Design and generation of reverse genetics-derived NiV. (**A**) A phylogenetic tree based on full-length genomic sequences of various NiV. Sequence alignment was performed with ClustalW, using the MEGA Version 7.0 software package^68^. The trees were constructed using the neighbor-joining algorithm with 1000 bootstrap replicates. The reproducibility of relevant nodes is shown as percentages. GenBank accession numbers for each sequence are listed below. The tree is drawn to scale, with branch lengths corresponding to the evolutionary distances used to infer the phylogenetic tree. (**B)** Similarity plot of nucleotide genome alignment using NiV/BD/HY/2004/RA as the query sequence were generated with Simplot v 3.5.1^71^. Prior to the analysis genome sequences were aligned with the Clustal Omega version 1.2.4^69^. After gaps were stripped the alignment comprised 18, 246 nucleotides (nt) and was assessed using a window of 500 nt and a step of 50 nt. The strains included were NiV/IN/HU/2007/FJ (blue), NiV/BD/HU/2008/Manikgonj (red), NiV/MY/HU/99/C2 (purple), and HeV/AU/HO/1997(green). (**C)** Schematic depictions of the constructed full-length cDNA expression constructs, pSPNiV-M, pSPNiV-B, and the recombinant NiV EGFP-expressing constructs, pSPNiV-M/G-P2A-EGFP, pSPNiV-M/EGFP, pSPNiV-B/G-P2A-EGFP, and pSPNiV-B/EGFP are shown. Non-coding regions and untranslated regions are depicted as narrow rectangles, and coding regions are represented as arrows with their respective gene names (N, P, M, F, G, and L) and are grouped by colour (blue: transcriptase complex, yellow: matrix protein, purple: attachment and fusion glycoproteins, green: inserted enhanced green fluorescent protein, EGFP). The dashed vertical lines indicate the unique restriction sites used to assemble the 4 cDNA fragments (**A**–**D**) that spanned each genome. The two mutations (S207L and G252D) introduced into the fusion protein of the pSPNiV-B are indicated with a black arrowhead. Additional structural features included are polII, the CMV promoter sequence for RNA polymerase II; HamRz, hammerhead ribozyme sequence; HdRz, hepatitis delta virus ribozyme; ϕ, beta globin transcription terminator; 3′, 3′ leader; 5′, 5′ leader; and P2A, porcine teschovirus 2A protease cleavage peptide-encoding sequence. The following short-forms were used in the strain names: MY, Malaysia; BD, Bangladesh; IN, India; HO, horse; PI, pig; HU, human; BA, bat. The GenBank accession numbers for the strains used include: NV/MY/PI/99/UM-0128 (AJ564623), NV/MY/PI/99/VRI-2794 (AJ564621), NiV/MY/HU/99/C1 (AY029767), NiV/MY/HU/1999/C2 (AY029768), NiV/MY/PI/99/VRI-1413 (AJ564622), NiV/BD/HU/2004/RA (AY988601), NiV/IN/HU/2007/FJ (FJ513078), NiV/BD/HU/2008/Manikgonj (JN808857), NiV/BD/HU/2008/Rajbari (JN808863), and HeV/AU/HO/1997 (AF017149.3).

We initially constructed a phylogenetic tree to assess the relatedness of NiV strains from outbreaks that had occurred in the time spanning 1998–2016 and to identify which strains of NiV-M and NiV-B were well-conserved with circulating strains, were available, and/or had full genomic sequences, including the genomic termini, deposited into GenBank (Fig. 1A). We then performed a Simplot analysis of the two candidate strains, NiV/MY/HU/1999/C2, also known as UMMC2 (accession no. AY029768), isolated from a throat secretion sample obtained in Malaysia in 1999^51^ that was available as a deposited sample in our institutional repository, and NiV/BD/HU/2004/RA, also known as SPB200401066 (accession no. AY988601), isolated from a human oropharyngeal swab sample obtained in Rajbariin in Bangladesh in 2004^11^ that had also been used in a previous pathogenicity study^38^. We found that NiV/MY/HU/1999/C2 and NiV/BD/HY/2004/RA were highly conserved with viruses isolated from Malaysia and from Bangladesh/India, respectively (Fig. 1B), and therefore were suitable strains for the construction of NiV-M and NiV-B reverse genetics systems.

The infectious cDNA clone of NiV/MY/HU/1999/C2 was generated by inserting the full-length anti-genomic cDNA into the high copy number plasmid, pSP-NA, under the control of the eukaryotic RNA polymerase II cytomegalovirus immediate early promoter (polII), using a strategy that we previously reported^52^. To generate the correct genomic termini upon polII transcription, a self-cleaving hammerhead ribozyme (HHrbz) and a hepatitis delta virus ribozyme (HdRz) (Supplementary Fig. S1) followed by a beta globin transcription terminator were inserted at the 3′ and 5′ ends of the anti-genomic sequence, respectively (Fig. 1C). The infectious cDNA clone of NiV/BD/HY/2004/RA was similarly constructed except the anti-genomic cDNA was chemically synthesized as oligonucleotides and sequentially assembled. Further, two amino acid substitutions were introduced into the F protein coding sequence, S207L and G252D, when initial efforts to rescue an infectious clone that exactly matched the deposited GenBank sequence (accession no. AY988601) failed, and it was noted that amino acids 207 and 252 of the F protein were conserved as leucine (L) and aspartic acid (D), respectively, in all of the other deposited sequences for HeV and NiV. The full-length viral genome cDNA insert was found to be highly stable in the high copy number, pSP-NA backbone plasmid when transformed bacteria were grown at 32 °C.

Recombinant NiV-M and NiV-B cDNA constructs expressing EGFP were constructed as above, except that an EGFP coding sequence was inserted downstream of the G protein coding region, either in frame with G with a P2A cleavage site inserted between G and EGFP (pSPNiV-M/G-P2A-EGFP and pSPNiV-B/G-P2A-EGFP) or as a separate gene with the G-L intergenic sequence duplicated and placed between G and EGFP (pSPNiV-M/EGFP and pSPNiV-B/EGFP) prior to the assembly of the recombinant full-length infectious clone constructs (Fig. 1C). We chose to place the EGFP ORF between the G and L genes, rather than between the P and M genes, with the goal of striking a balance between preserving the viral polar transcription gradient and replication kinetics and achieving a high level of EGFP expression.

Infectious virus that corresponded to the deposited sequences for NiV/MY/HU/1999/C2 and NiV/BD/HY/2004/RA (with the exception of the noted amino acid substitutions) and the recombinant EGFP expressing NiV were recovered by co-transfection of pSP-NiV-M, pSP-NiV-B, pSP-NiV-M/EGFP, pSP-NiV-M/G-P2A-EGFP, pSP-NiV-B/EGFP, or pSP-NiV-B/G-P2A-EGFP, and the corresponding helper plasmids, expressing the N, P, or L proteins of NiV-B into HEK 293 GripTite (293GT) or BHK-21 cells (used for the rescue of rgNiV-B/EGFP since the rescue efficiency was found to be greater in these cells). The amino acid identity between the N, P, and L proteins of NiV-M and NiV-B are 98%, 92%, and 98%, respectively, and many of the non-conserved amino acids are physio-chemically conserved and the proteins of the two strains are functionally compatible with either genome. This was demonstrated by our ability to efficiently rescue either virus with the NiV-B helper plasmids. After two passages in Vero E6 cells the rescued wild type viruses, rgNiV-M and rgNiV-B, reached a titer of 1.2 × 10^7^ and 7.9 × 10^7^ TCID_50_/ml, respectively, and the EGFP expressing viruses, rgNiV-M/EGFP, the rgNiV-M/G-P2A-EGFP, and rgNiV-B/G-P2A-EGFP, reached a titer of 2.1 × 10^7^, 1 × 10^8^, and 3.2 × 10^7^ TCID_50_/ml, respectively. Previous studies have used the quantification of EGFP-positive rescue cells by flow cytometry as a measure for paramyxovirus rescue efficiency^53^; however, we found that not all the EGFP-positive, rescue cell-containing wells would progress to show discernable CPE. To measure the rescue efficiency of this reverse genetics system we, therefore, rescued rgNiV-M/EGFP and rgNiV-B/EGFP in BHK-21 cells and counted the number of wells that displayed positive EGFP expression and went on to develop CPE (Supplementary Fig. S1). The rescue efficiency was found to be 75% for both rgNiV-M and rgNiV-B.

### *In vitro* characterization of rgNiV-M and rgNiV-B viruses

**Figure 2:**
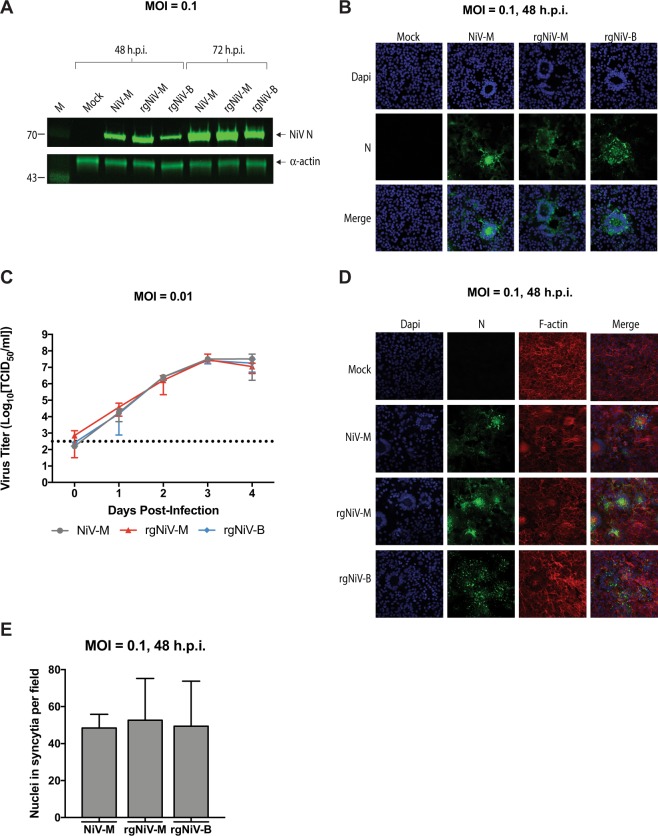
Characterization of reverse-genetics derived NiVs. (**A**) Western blot analysis of the NiV nucleoprotein (N) from infected cell lysates. Vero E6 cells were mock-infected, infected with NiV-M isolate, rgNiV-M, or rgNiV-B (MOI = 0.1), and lysates were harvested 48 hours later. Lysates were subjected to SDS-PAGE followed by Western blotting and were probed a monoclonal antibody against the N protein of NiV-M. α-actin served as a loading control. The lysates were run on duplicate SDS-PAGE gels and the respective Western blots were stained for NiV N or α-actin. Protein standards are in lane 1 and the band sizes are indicated in kilodaltons. (**B)** Immunofluorescence analysis of Vero E6 cells that were mock-infected, infected with NiV-M, rgNiV-M, or rgNiV-B. Cells were fixed 48 hours after infection and were subsequently stained with monoclonal antibody against the N protein and visualized by confocal microscopy. (**C**) Growth kinetics of NiV-M and reverse genetics-derived NiV. Vero E6 cells were infected with NiV-M, rgNiV-M, or rgNiV-B at an MOI of 0.01. Supernatants were collected at the indicated days post-infection and titrated by standard TCID_50_ analysis in VeroE6 cells. The mean and standard deviations from three biological replicates are shown. The dashed line indicates the limit of detection for the assay. (**D**) Fusogenicity of wildtype NiV-M and reverse-genetics derived NiVs. Vero E6 cells were mock-infected, infected with NiV-M, rgNiV-M, rgNiV-B (MOI = 0.1) and cells were fixed 48 hours later. To reveal the presence of infected cells with multinucleated syncytia, fixed cells were stained with Mab against the N protein (green), phalloidin to detect F-actin (red), allowing for the demarcation of individual cells, and DAPI to detect nuclei (blue). (**E**) Cells with three or more nuclei within an N protein positive cell were counted in five different fields (magnification, x40) per treatment. Bars indicate mean values and error bars indicate s.d. Scale bars, 2 mm in b and d. Statistical differences were not significant as determined by the student T test (p > 0.05). The limit of detection for the TCID_50_ assays was 10^2.5^ TCID/ml.

In order to characterize the reverse genetics-derived viruses, Vero E6 cells were either mock-infected or infected with the NiV-M clinical isolate (NiV-M), rgNiV-M, or rgNiV-B at an MOI of 0.1, and cell lysates were harvested at 42 hours or 72 hours post-infection (hpi). A clinical isolate of NiV-B was not available to us at the time these studies were performed. Western blotting with a monoclonal antibody against the N protein (F45G2)^54^ revealed a distinct band of the expected size of approximately 70 kDa in the NiV-M and NiV-B infected cell lysates, which was absent in lysates derived from the mock-infected, control cells (Fig. 2A, Supplementary Fig. S1). At 48 and 72 hpi the expression of N in NiV-M-infected cell lysates was comparable to the expression of N in rgNiV-M-infected cell lysates, while expression of N in rgNiV-B-infected cell lysates was markedly lower (Fig. 2A). Further experimentation will be required to determine whether this is reproducible and actually reflects the relative levels of N expression or perhaps differential detection with the MAb against the N protein of NiV-B. As replication continued between 48 and 72 hpi there was an expected concomitant increase in the level of N detected for each virus (Fig. 2A). Immunofluorescence analysis of VeroE6 cells either mock-infected or infected with the NiV-M isolate, rgNiV-M, or rgNiV-B at an MOI of 0.1 revealed comparable levels of N expression and a similar cellular distribution of N in infected cells, with fluorescence observed in the perinuclear and cytoplasmic regions of infected cells that were primarily located in large, multi-nucleated syncytium. The mock-infected cells showed no N staining and no cytopathic effect (CPE), including a lack of any syncytia (Fig. 2B).

The replication kinetics of the NiV-M, rgNiV-M, and rgNiV-B were compared in Vero E6 cells (MOI = 0.01). Supernatants from infected cells were harvested at 0, 24, 48, 72, and 96 hpi, and infectious virus titers were determined by TCID_50_ assay (Fig. 2C). At all the time points evaluated supernatants from rgNiV-M and rgNiV-B-infected cells had comparable titers, and at no time point was the difference statistically significant by two-way ANOVA with p > 0.8514. Further, rgNiV was found to replicate with the same kinetics as the NiV-M isolate, and all three viruses reached similar titers by 72 hpi, exceeding an average of 1 × 10^7^ TCID_50_/ml (Fig. 2C).

The ability of NiV-M, rgNiV-M, and rgNiV-B to form syncytium in infected cells were assessed by immunofluorescence analysis in Vero E6 cells infected at an MOI of 0.1 (Fig. 2D,E). Syncytium formation was quantified by determining the average number of nuclei per microscopic field of view (40x magnification, 5 fields total) that were contained within NiV N expressing cells with greater than three nuclei at 48 hpi. All three viruses displayed similar levels of syncytium formation with no significant difference (student T test, p > 0.05) (Fig. 2E).

### *In vivo* characterization of rgNiV-M and rgNiV-B

**Figure 3:**
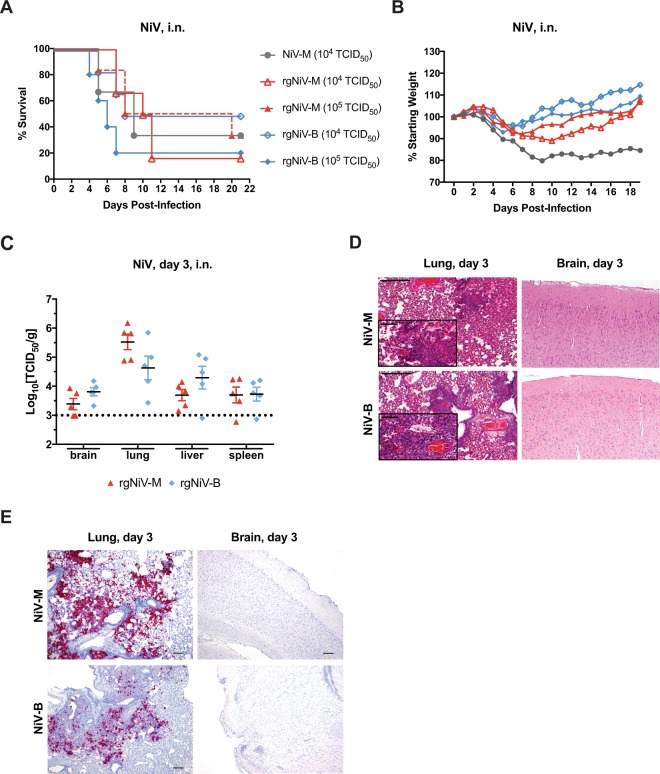
NiV infection of Syrian hamsters. Groups of five-to-six-week week-old female hamsters were inoculated with 1 × 10^4^ or 1 × 10^5^ TCID_50_ of rgNiV-M (n = 6), 1 × 10^4^ or 1 × 10^5^ TCID_50_ rgNiV-B (n = 6) or 1 × 10^4^ TCID_50_ NiV-M (n = 3) by the intranasal route (i.n.). Hamsters were observed daily to assess the clinical signs and survival. The results from two separate experiments were pooled, and the Kaplan-Meier curves representing survival data (**A**) and the weight loss (**B**) represent combined data. The log rank test of the Kaplan-Meier curves yielded P values greater than 0.05 and were not significant. At day three post-infection, the indicated tissues were harvested, weighed, homogenized, and analyzed by TCID_50_ assay (**C**). Histopathological analysis (H&E staining) of the lung and brain (**D**) were performed. Areas of hemorrhage and infiltration of inflammatory cells are shown. Evaluation of lung and brain tissue samples for the presence of NiV target RNA using *in situ* hybridization by RNAScope assay (**E**). Multifocal areas of intense viral RNA staining in a lung section (red, left panels). Brain sections were negative for the presence of viral RNA (right panels). Scale bars, 500 µM (**D**), 50 µM (inserts) (**D**), and 200 µm (**E**). The limit of detection for both TCID_50_ assays was 10^3^ TCID_50_/g.

In order to assess whether or not the reverse genetics-derived viruses were pathogenic when administered *in vivo*, we compared NiV-M, rgNiV-M, and rgNiV-B in the Syrian hamster model of infection^4^. Five-to-six-week old female Syrian golden hamsters (*Mesocricetus auratus*) were challenged by the intranasal route (i.n.) with 10-fold serial dilutions (10^1^ to 10^5^ TCID_50_) of NiV-M, rgNiV-M, and rgNiV-B. Hamsters challenged with any of the three viruses exhibited similar clinical signs that included hunched posture, ruffled coat, and at the later stages of infection reduced activity, labored breathing, and neurological manifestations that included tremors, hind-limb paralysis, and seizures. The clinical signs associated with the respiratory stage of illness (including labored breathing) occurred between 4–8 dpi, whereas the severe neurological manifestations that required humane euthanasia consistently occurred at 9 dpi or later. Some animals exposed to each of of the three viruses (one or no animals per LD_50_ dosage group) were apparently not infected, showing no clinical signs for the duration of the study. Unfortunately, serum was not collected at the time of euthanasia from the surviving animals, therefore, we were subsequently unable to determine whether the animals that did not exhibit disease had been exposed to virus and seroconverted. This is something that we will need to address in future studies in the intranasal infection of hamsters with these viruses. The median (50%) lethal dose (LD_50_) for NiV-M, rgNiV-M, and rgNiV-B was 3.2 × 10^3^ TCID_50_, 2.2 × 10^3^ TCID_50_, and 3.5 × 10^2^ TCID_50_, respectively; however, we note that the Kaplan-Meier survival curves at a dose of 10^4^ TCID_50_ were not significantly different between NiV-M and rgNiV-M (log rank test, p = 0.90) or between rgNiV-M and rgNiV-B (log rank test, p = 0.55) (Fig. 3A). The Kaplan-Meier survival curves for all doses are provided for rgNiV-M and rgNiV-B (Supplementary Fig. S1). The average time to death for animals that succumbed at a 10^4^ TCID^50^ dose were 7, 9.2, and 6.7 days post-infection (dpi) for animals challenged with NiV-M (n = 3), rgNiV-M (n = 6), and rgNiV-B (n = 6), respectively, and the differences were not significant between NiV-M and rgNiV-M (student T test, p = 0.29) nor between rgNiV-M and rgNiV-B (student T test, p = 0.12) (Fig. 3A). The hamsters that were challenged with 10^5^ TCID_50_ of rgNiV-M (n = 6) or rgNiV-B (n = 5) had Kaplan-Meier survival curves that were not significantly different from one another (log rank test, p = 0.22), and the average time to death among the animals that succumbed did not differ significantly (student T test, p = 0.114) (Fig. 3A).

Weight loss for the majority of hamsters exposed to 10^4^ TCID_50_ (NiV-M, rgNiV-M, rgNiV-B) or 10^5^ TCID_50_ (rgNiV-M or rgNiV-B) progressed gradually from 3 dpi onwards until the animals had reached the predefined scoring endpoint or began to rebound and gain weight (Fig. 3B), while a single animal exposed to 10^4^ TCID_50_ of rgNiV-B did not lose any weight for the duration of the study.

Next, we assessed the infectious virus titers in the brain, lung, liver, and spleen tissue of NiV-M, rgNiV-M, and rgNiV-B-infected hamsters (10^5^ TCID_50_) at 3 dpi (Fig. 3C). Infectious rgNiV-M or rgNiV-B was detected in all of the organs with the highest titers seen in the lung for hamsters challenged with either virus at 10^5.5^ TCID_50_/g and 10^4.6^ TCID_50_/g for rgNiV-M and rgNiV-B-infected hamsters, respectively. The difference between the viral load in the brain, lung, liver, or spleen of rg-NiV-M or rgNiV-B-infected hamsters were not statistically significant (student T test, p = 0.150, 0.182, 0.098, 0.863, respectively). Tissues from infected hamsters were examined histologically (Fig. 3D). Pathology was observed in the lungs for both rgNiV-M and rgNiV-B infections, which consisted of mild to moderate multifocal, bronchointerstitial pneumonia. Infiltration of macrophages and neutrophils was evident in localized areas of tissue, as well as some necrosis, edema and fibrin deposits (Fig. 3D, left panels). Small to medium areas of acute hemorrhage were also seen in some sections. Brain tissues were also examined but pathology was not evident in any of the infected hamsters sacrificed at 3dpi (Fig. 3D, right panels). Brain and lung sections from rgNiV-M and rgNiV-B-infected hamsters were examined using *in situ* hybridization by RNAscope assay (n = 5). Viral RNA was detected within all the lung sections but varied from a small amount ( < 10 cells) to a large amount involving over 75% of the section (Fig. 3E, left panels). No viral RNA was detected in any of the brain sections examined (Fig. 3E, right panels).

### Development of rgNiV-M/EGFP and rgNiV-B/EGFP-based assays

**Figure 4:**
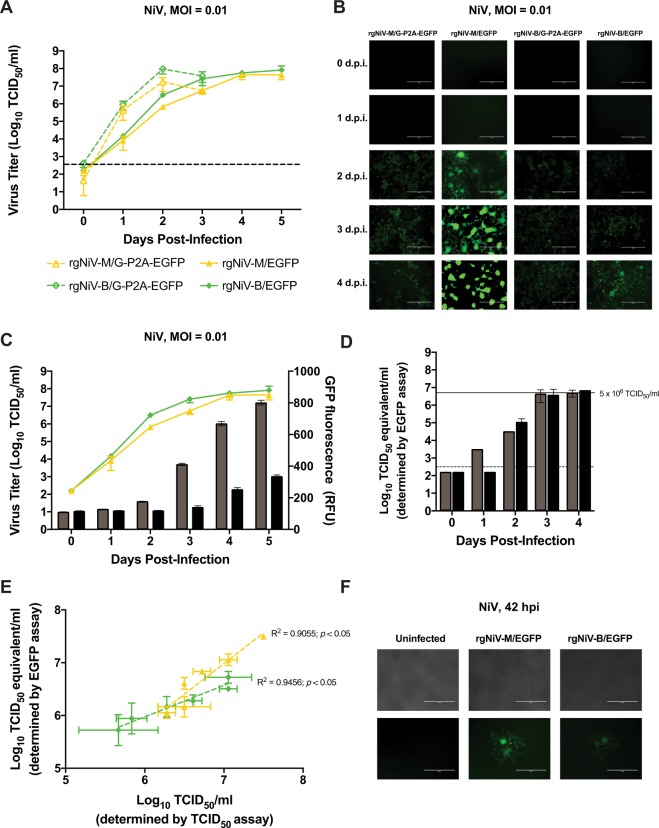
Characterization of recombinant, EGFP-expressing NiVs. (**A**) Growth kinetics of recombinant NiV expressing EGFP. Vero E6 cells were infected with rgNiV-M/G-P2A-EGFP, rgNiV-M/EGFP, rgNiV-B/G-P2A-EGFP, or rgNiV-B/EGFP at an MOI of 0.01. Supernatants were collected at the indicated days post-infection and titrated by standard TCID_50_ analysis in VeroE6 cells. The mean and standard deviation from three biological replicates are shown. The dashed line indicates the limit of detection for the assay. (**B**) Epifluorescence microscopy of cells infected with EGFP-expressing NiVs. Vero E6 cells were infected with rgNiV-M/G-P2A-EGFP, rgNiV-M/EGFP, rgNiV-B/G-P2A-EGFP, or rgNiV-B/EGFP at an MOI of 0.01 and representative images were taken of EGFP expression. (**C**) Quantification of EGFP expression in rgNiV-M/EGFP or rgNiV-B/EGFP-infected cells. EGFP activity resulting from the infection of Vero E6 cells with rgNiV-M/EGFP (grey bars) or rgNiV-B/EGFP (black bars) at a MOI of 0.01 was measured daily for five days using a fluorescence-based microplate reader and plotted against viral titer in rgNiV-M/EGFP (yellow line or rgNiV-B/EGFP-infected supernatants (green line), as described above (same viral titer data as panel A). (**D**) EGFP-based TCID_50_ assay. A TCID_50_ assay using EGFP as a readout was carried out on rgNiV-M/EGFP (grey bars) or rgNiV-B/EGFP samples (black bars) of known concentration diluted to 5 × 10^6^ TCID_50_/ml, indicated by a solid line. Fluorescence foci were identified on the indicated days post-infection and the TCID_50_/ml equivalent titers of the input were calculated. The dashed line indicates the limit of detection for the assay. Bars indicate mean values and error bars indicate s.d. from four replicates. (**E**) Comparison of EGFP-based titration assay and conventional TCID_50_ assay. Samples containing two-fold dilutions of rgNiV-M/EGFP (yellow line) or rgNiV-B/EGFP (green line) were titrated in duplicate by the EGFP-based titration assay at three dpi and a conventional TCID_50_ assay at five dpi and were plotted on the same graph. The dashed line indicates the line of best fit, and the R^2^ value is given. (**F**) Fluorescence foci in rgNiV-M/EGFP or rgNiV-B/EGFP-infected cells. Vero E6 cells were mock-infected or infected with the respective viruses at an MOI of 0.01 and representative images were taken of EGFP expression at 48 hpi. Bars indicate mean values and error bars indicate sd. The limit of detection for the TCID_50_ assays was 10^2.5^ TCID/ml.

In order to characterize the recombinant NiV expressing EGFP we compared the replication kinetics of rgNiV-M/EGFP, rgNiV-M/G-P2A-EGFP, rgNiV-B/EGFP, and rgNiV-B/G-P2A-EGFP in Vero E6 cells (MOI = 0.01) (Fig. 4A). Infected cell culture supernatants were harvested daily until the cell monolayer was destroyed (0, 24, 48, and 72 hpi and 0, 24, 48, 72 96, and 120 hpi for rgNiV-M/G-P2A-EGFP/ rgNiV-B/G-P2A-EGFP and rgNiV-M/EGFP/ rgNiV-B/EGFP, respectively). Viral titers were then determined by a standard TCID_50_ assay. The replication kinetics of rgNiV-M/EGFP and rgNiV-B/EGFP were significantly delayed compared to rgNiV-M/G-P2A-EGFP and rgNiV-B/G-P2A-EGFP at 24 and 48 hpi in Vero E6 cells (MOI = 0.01) (one-way ANOVA, p = 0.047 and 0.048, respectively). All four viruses reached a similar titer by 72 hpi (Fig. 4A). It was noted that the CPE in the rgNiV-M/EGFP and rgNiV-B/EGFP-infected cells at 72 hpi did not yet involve the full monolayer (data not shown), and complete CPE was not observed in cells infected by these viruses until 120 hpi. The endpoint titer at 120 hpi in rgNiV-M/EGFP and rgNiV-B/EGFP-infected cells were similar to those reached in rgNiV-M/G-P2A-EGFP and rgNiV-B/G-P2A-EGFP-infected cells at 72 hpi (one-way ANOVA, p = 0.129). The intensity of EGFP expression was visualized by epifluorescence microscopy 0, 24, 48, and 72, and 96 hpi (Fig. 4B). While the EGFP signal increased gradually as the infection progressed for all three viruses, the EGFP signal from the rgNiV-M/EGFP-infected cells was considerably brighter than the signal from the rgNiV-M/G-P2A-EGFP or rgNiV-B/G-P2A-EGFP-infected cells, and the signal from rgNiV-B-EGFP infected cells was noticeably stronger than cells infected with either P2A-containing construct. It is worth noting that rgNiV-M/EGFP and rgNiV-B/EGFP express EGFP as an independent open reading frame while rgNiV-B/G-P2A-EGFP and rgNiV-M/G-P2A-EGFP express EGFP from a self-cleaving C-terminus G-fusion protein. Due to the greater EGFP signal output in infected cells, subsequent experiments were performed using rgNiV-M/EGFP and rgNiV-B/EGFP.

The intensity of the EGFP fluorescence signal of rgNiV-M/EGFP and rgNiV-B/EGFP-infected Vero E6 cells (MOI = 0.01) was then measured on a microplate reader daily up to 5 dpi and the obtained values were plotted along with the corresponding viral titers in cell culture supernatants as determined by TCID_50_ assay (Fig. 4C). The EGFP fluorescence signals were passively obtained, without interfering with the viral replication kinetics, and reached a plateau of 801 ± 14.9 and 335 ± 9.2 relative fluorescence units (RFU) in rgNiV-M/EGFP and rgNiV-B/EGFP-infected cells at 5 dpi, respectively (Fig. 4C). Further, we serially passaged both rgNiV-M/EGFP and rgNiV-B/EGFP a total of 4 times in VeroE6 cells, resulting in passage 6 (P6) viruses, and EGFP expression was observed by epifluorescence microscopy with no apparent reduction in EGFP signal intensity (Supplementary Fig. S1).

In order to assess the suitability of rgNiV-M/EGFP and rgNiV-B/EGFP for use in an EGFP fluorescence foci-based TCID_50_ assay, we visually observed Vero E6 cells infected with serial 10-fold dilutions of an experimental sample (four biological replicates, done in triplicate), containing rgNiV-M/EGFP or rgNiV-B/EGFP of known concentration daily from 0 to 4 dpi, scoring wells containing EGFP fluorescence foci as positive and wells with no fluorescent foci as negative, and subsequently performed a TCID_50_ analysis (Fig. 4D). EGFP-based TCID_50_ calculations were found to reach the known input titer at 3 dpi, a full two days earlier than the conventional TCID_50_ calculation that was read at 5 dpi once large syncytia and advanced CPE are observable from the low titer at the highest infectious dilution. We noted that some syncytia could be identified by bright-field microscopy as early as 24 hpi (and thus virus-positive wells could be identified); however, we have found that reading the conventional TCID_50_ assay earlier than 4 or 5 dpi resulted in an underestimation of the titer (data not shown). Further, prior to 3 dpi syncytia were present that could be identified by EGFP expression but were not always readily discernable by bright-field microscopy at that time (data not shown).

Next, experimental samples containing rgNiV-M/EGFP or rgNiV-B/EGFP of known concentrations were serially diluted 2-fold and the resulting samples were subsequently evaluated by both the EGFP-based TCID_50_ and the conventional TCID_50_ assay in triplicate at 3 and 5 dpi, respectively, and were plotted together on the same graph (Fig. 4E). A linear relationship was found to describe both data sets with a slope of 1.16 ± 0.19 and 0.60 ± 0.071 with R^2^ values of 0.9055 (*p* < 0.05) and 0.9456 (*p* < 0.05) for the rgNiV-M/EGFP and rgNiV-B/EGFP dilution series, respectively. Infection of Vero E6 with rgNiV-M/EGFP or rgNiV-B/EGFP (MOI = 0.01) resulted in clearly distinguishable fluorescent foci, corresponding to developing syncytia, as early as 2 dpi, well before the total number of NiV plaques/syncytia are easily discernible (Fig. 4F).

### Evaluation of neutralizing antibodies with rgNiV-M/EGFP

**Figure 5:**
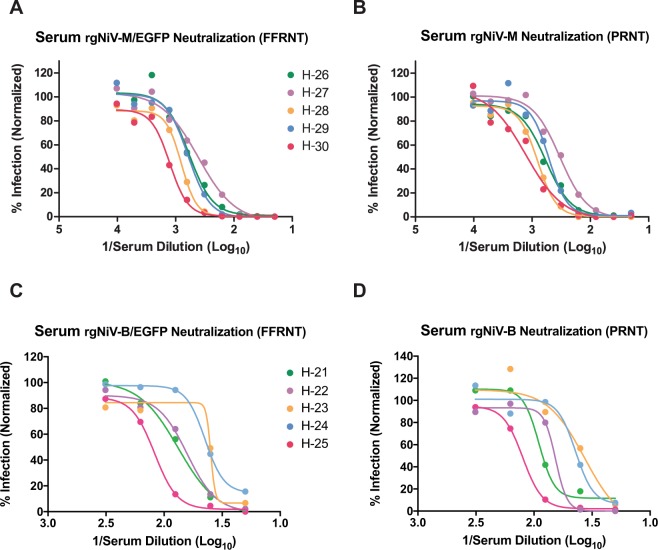
Evaluation of neutralizing antibodies with rgNiV-M/EGFP or rgNiV-B/EGFP. Increasing concentrations of serum from hamsters exposed to attenuated rgNiV-M or rgNiV-B were incubated with 50 pfu of rgNiV-M/EGFP (**A**), rgNiV-M (**B**), rgNiV-B/EGFP (**C**) or rgNiV-B (**D**) and inhibition was assessed with a fluorescence focus reduction neutralization test (FFRNT) (**A**,**C**) and a conventional plaque reduction neutralization test (PRNT) (**B**,**D**). Regression analysis and graphing was performed with Prism statistical software (GraphPad).

Next, we sought to evaluate the effectiveness of rgNiV-M/EGFP and rgNiV-B/EGFP -based fluorescence focus reduction neutralization tests (FFRNT) compared to a conventional plaque reduction neutralization tests (PRNT) to detect the presence of neutralizing antibodies and to determine neutralizing antibody titers in the serum of hamsters that had survived exposure to attenuated rgNiV-M (Fig. 5A,B) or rgNiV-B (Fig. 5C,D). While the FFRNTs were read 2 days earlier than the PRNTs (3 dpi versus 5 dpi), the rgNiV-M/EGFP-based FFRNT_90_ titers for the NiV-M exposed hamster serum samples matched the PRNT_90_ titers for all five experimental samples tested, and the FFRNT_50_ and PRNT_50_ titers matched for 3 out of the 5 samples tested, differing by one dilution in both instances (FFRNT_50_/PRNT_50_ = 1:320/1:640 and 1:1280/1:640). Further, the rgNiV-B/EGFP-based FFRNT_90_ titers for the NiV-B exposed hamster serum samples matched the PRNT_90_ titers for three out of five experimental samples tested, differing by one dilution in both instances (FFRNT_90_/PRNT_90_ = 1:320/1:160 and 1:320/1:160). The rgNiV-B/EGFP-based FFRNT_50_ titers for the NiV-B exposed hamster serum samples matched for 4 out of the 5 samples tested, differing by one dilution (FFRNT_50_/PRNT_50_ = 1:80/1:160).

## Discussion

In this study, we describe a highly efficient pol II-driven reverse genetics system for NiV-M and NiV-B (Fig. 1). The reverse genetics-derived virus, rgNiV-M, constructed from cDNA derived from a strain isolated in Malaysia was indistinguishable *in vitro* from the corresponding clinical isolate when applied to Vero E6 cells, expressing a comparable level of the nucleocapsid protein, replicating with similar growth kinetics, and exhibiting a similar level of fusogenicity (Fig. 2). The reverse genetics-derived virus, rgNiV-B, was rescued from a full-length cDNA expression construct that was chemically synthesized based on the sequence of a human strain isolated in Bangladesh (Fig. 1). *In vitro*, rgNiV-M and rgNiV-B replicated with similar growth kinetics and exhibited a similar level of fusogenicity (Fig. 2). Both reverse genetics-derived viruses were pathogenic when administered to Syrian hamsters, resulting in weight loss, pulmonary pathology, severe neurological signs, and lethality (Fig. 3). The Kaplan-Meier survival curves and the average time to death were not significantly different when hamsters were exposed to rgNiV-M versus rgNiV-B or to rgNiV-M versus the corresponding NiV-M clinical isolate. Infectious virus was detected in the brain, lung, liver, and spleen in hamsters infected with each of of the reverse genetics-derived viruses, and pathology was noted in the lung tissue (Fig. 3). We also constructed recombinant NiV-M, rgNiV-M/EGFP, and NiV-B, rgNiV-B/EGFP, that to our knowledge are the first rNiV to expresses EGFP from an open reading frame (ORF) inserted between the G and L genes, and we developed a rapid and sensitive EGFP fluorescence foci-based TCID_50_ assay for the detection of NiV infection and the determination of NiV titers *in vitro* (Fig. 4). We further demonstrated in a proof-of-principle study the suitability of rgNiV-M/EGFP and rgNiV-B/EGFP for *in vitro* screening for neutralizing antibodies (Fig. 5).

This study constitutes the first report of a full-length, henipavirus antigenomic DNA (cDNA) clone rescued under the control of cellular pol II (Fig. 1) and a useful update to the NiV reverse genetics system. The first generation reverse genetics system reported for NiV-M relied on expression of the bacteriophage T7 polymerase (T7pol) from a recombinant vaccinia virus to drive expression of NiV cRNA from a T7 promoter^41^, whereas successive recombinant henipaviruses have employed co-transfection with a T7pol expression construct^39,42^ or were rescued in a cell line that stably expresses T7pol^43^. Various viruses of the *Mononegavirales* order, including rabies virus (RV), infectious Borna disease virus (BDV), Newcastle disease virus (NDV), and measles virus (MV), the latter two of which are members of the *Paramyxoviridae* family along with henipaviruses, were found to be rescued with greater efficiency when cRNA was expressed from a PolII promoter rather than from a T7 promoter^55,56^. Further, a NiV-M mini genome rescue system driven by cellular DNA dependent RNA polymerase I (polI) was shown to express a higher level of expression of a reporter gene compared to a T7-driven mini genome rescue system^57^. An additional benefit of the pol II-driven reverse genetics system is that it eliminates the need to co-transfect cells with a T7pol expression construct or to construct and/or maintain a stable cell line expressing T7pol.

The ability to rescue negative sense RNA viruses under the control of a polII promoter does not appear to be negatively impacted by the size of the genome^58^. For example, we have successfully rescued the Makona variant of Ebola virus that is 18,959 nt in length^52^ as well as the viruses reported in this study, rgNiV-M and rgNiV-B, that are 18246 and 18252 nt in length, respectively. It remains unclear which cellular mechanism(s) direct the export of the viral cRNA from the nucleus; however, based on our findings, as well as those from other studies that are cited above, nuclear export of unspliced viral cRNA does occur at a high enough frequency to support efficient viral rescue. The presence of the self-cleaving hammerhead ribozyme sequence at the 5′ end of the genome and the hepatitis delta virus ribozyme sequence at the 3′ end of the genome direct the removal of the 5′-cap and the poly(A) tail of the cRNA, which may inhibit unwanted splicing of the viral cRNA and thereby increase the rate of export of full-length cRNA from the nucleus.

An additional technical improvement reported in this work is the use of a high-copy plasmid, a modified pSP72 vector, to house the full-length viral cDNA and the use of conventional competent *Escherichia coli* (*E*. *coli*) cells for plasmid propagation, which greatly simplified the process of genetically modifying the viral genome. In contrast, a modified low copy number plasmid, pBR322, was used as the backbone for a previously reported infectious clone for NiV-M^47,59^. We found that the full-length viral genome cDNA inserts were highly stable in all the reported constructs when bacteria were grown at 32 °C (data not shown). Similarly, a recombinant NiV cDNA cloned into pcDNA3.1(−) was reported to be stable when plasmids were grown at 30 °C in Stbl2 *E*. *coli* cells that are specially designed to allow the cloning of unstable inserts^42^.

The rescue efficiency for the pol II-driven reverse genetics system reported in this work was found to be 75% (Supplementary Fig. S1), measured as the number wells that displayed positive EGFP expression and produced visible CPE, representing one or more rescue events in the well, out of the total number wells transfected with either pSPNiV-M/EGFP or pSPNiV-B/EGFP and the three helper plasmids. A previous study by Beaty *et al*. in 2017 included a useful summary of the rescue efficiencies of the various paramyxovirus reverse genetics systems that have been reported in the literature^53^. Although the rescue efficiency was measured differently in either study, we estimate that our rescue efficiency would be comparable to the 10 rescue events/10^5^ cells reported in that work for a T7-driven NiV-M since greater than 20 EGFP positive cells were visualized in each 6-well plate at 48 hpi (data not shown).

We also explored the possibility and utility of generating recombinant NiV that express a marker gene inserted between the G and L genes (Fig. 1). The EGFP-expressing recombinant NiVs, rgNiV-M/EGFP and rgNiV-B/EGFP with EGFP inserted as an ORF that is flanked by duplications of the 5′ non-coding region (NCR) of L and the 3′ NCR of G (Fig. 1), proved to replicate with delayed growth kinetics, compared with rgNiV-M/G-P2A-EGFP and rgNiV-B/G-P2A-EGFP, both of which express EGFP as a self-cleaving C-terminus G-fusion protein (Figs 1 and 4). We suggest that the ORF insertion and/or the duplication of the G-to-L intergenic region may have resulted in reduced expression of the polymerase protein, L, which would explain the observed delay in replication. Despite this, the EGFP signal detected in rgNiV-M/G-P2A-EGFP or rgNiV-B/G-P2A-EGFP-infected VeroE6 cells was greatly reduced compared to rgNiV-M/EGFP or rgNiV-B/EGFP-infected cells, and infection with rgNiV-M/EGFP resulted in much greater EGFP fluorescence than with rgNiV-B/EGFP for unknown reasons (Fig. 4B). Since there was a reasonably high EGFP signal in rgNiV-M/EGFP-infected cells we concluded that, while signal attenuation due to the polar NiV transcription gradient likely contributed, the low EGFP signal in rgNiV-B/G-P2A-EGFP and rgNiV-M/G-P2A-EGFP-infected cells likely resulted from inefficient P2A cleavage. Future rgNiV-B/G-P2A-EGFP and rgNiV-M/G-P2A-EGFP constructs could be designed to include an optimized P2A sequence, as was included in a recently reported reverse genetics system for NiV-M^50^ to determine if the EGFP-signal in rgNiV-B/G-P2A-EGFP and rgNiV-M/G-P2A-EGFP-infected cells could be improved. We conclude that this genomic position for the reporter gene insertion between G and L was not optimal for achieving high EGFP expression, although the levels of EGFP are sufficient for direct observation and identification of infected cells when EGFP is inserted as an independent ORF.

It is interesting to note that a previous study comparing NiV-M and NiV-B infection both *in vitro* in BHK-21 cells and *in vivo* in Syrian hamsters showed differences in the viral growth kinetics and pathogenicity between the two viruses, whereas our *in vitro* and *in vivo* data showed no significant differences between the two strains^60^. We observed comparable CPE, replication kinetics, and endpoint titers when Vero E6 cells were infected with either NiV-M or NiV-B (Fig. 2), whereas the previous study reported little CPE, delayed growth kinetics, and lower endpoint titers when BHK-21 cells were infected with NiV-B compared to NiV-M. We suggest that this finding warrants further study to determine if cellular factors in BHK-21 cells or differences in the strains used resulted in these different observations. Further, we found that there was no significant difference in time to death when hamsters were exposed to 10^5^ TCID_50_ of NiV-M versus NiV-B by the i.n. route of infection (Fig. 3), whereas the previous study reported a statistically significant delay in time to death when hamsters were exposed to 10^5^ TCID_50_ of NiV-B versus NiV-M by either an intraperitoneal (i.p.) or i.n. route of infection. Lastly, we detected similar infectious titers of NiV-M or NiV-B in the lung, brain, and spleen of infected hamsters at 3 dpi by an i.n. route (Fig. 3), whereas the previous study reported less NiV-B RNA than NiV-M RNA in the brain at 3 dpi following the exposure of hamsters to 10^5^ TCID_50_ of either virus by an i.p. route of infection. We suggest that the contrasting outcomes in the two studies following *in vitro* and *in vivo* infection with NiV-B versus NiV-M could be the result of differences in the viral populations present in the respective inoculums. The NiV-B inoculum used in the original study had been passaged 3 times in Vero E6, whereas the reverse genetics derived NiV-B used in the present study was passaged only twice in Vero E6 and is, therefore, less likely to have become attenuated. Alternatively, the NiV-B sequence that we used to synthesize the infectious clone, NiV/BD/HY/2004/RA (accession no. AY988601), may not be truly representative of an actual contiguous viral genomic sequence or may not represent the dominant variant within the viral population that was present in the oropharyngeal swabs from which the virus was isolated. Further, it is known that genetic variability in the population of viruses present in a clinical virus isolate itself can influence the pathogenicity of an isolate *in vivo*^61^. The clonal virus that we used in this study, removed from the viral population, may have increased virulence *in vivo*. Previous deep sequencing of NiV inoculums have revealed sequence differences between NiV-M and NiV-B isolates and their respective sequences in GenBank, and multiple coding changes were identified in a NiV-B P2 stocks compared to the reference sequence with GenBank Accession number AY988601 (that we used as the basis for our synthesized rgNiV-B construct) that included amino acids 207 and 252 of the F protein that we had to mutagenize to generate a rescuable virus^38^. It would be interesting to perform next-generation sequencing on the respective virus stocks to determine if there are differences in the viral populations that result in different outcomes in animal models of NiV infection, although at this time the clinical isolate of NiV-B is not available to us. Interestingly the NiV-B strain was found to be more pathogenic than the NiV-M strain in the African green monkey (AGM) model of NiV infection^38^, recapitulating what is observed in humans^12–15,25–27^. Therefore, the pathogenesis of NiV-B versus NiV-M observed in our hamster study more closely resembles what is observed in AGMs and humans than the previously described hamster pathogenesis study, where hamsters were either exposed to a NiV-M strain isolated from human brain tissue or to the isolate, NiV/BD/HY/2004/RA (accession no. AY988601), that was the basis for our synthetic construct rgNiV-B^60^.

The synthesis of NiV-B reported in this study as well as the synthesis of Cedar virus^40^, an apathogenic henipavirus, demonstrate a practical workflow and proof-of-principle for the rapid generation of infectious henipavirus when a full genomic cDNA sequence is available prior to the availability of a clinical isolate or when a virus isolate does not exist. While the spillover of only two henipaviruses, HeV and NiV, into humans has occurred with regularity, henipavirus RNA sequences have been detected in bats^62–64^, and anti-henipavirus antibodies have been identified in bats^62,65^, rats^66^, and humans in Cameroon, Africa^67^. NiV has been deemed a pandemic threat^35^, and should a novel pathogenic henipavirus spill over into humans, rapid dissemination of the full genomic sequence would allow BSL4 laboratories to synthesize and rescue the virus by reverse genetics to facilitate a rapid worldwide diagnostics and research response.

The availability of a reverse genetics system for NiV-B will have important implications for future studies, including the ability to explore the molecular basis for the pathogenic differences associated with NiV-M versus NiV-B infection in humans and the susceptibility of intermediate, amplifying animal hosts such as pigs. Further, a reverse genetics system for NiV-B will also facilitate the targeted screening and evaluation of therapeutics and candidate compounds against contemporary strains of NiV.

## Methods

### Ethical statement

All work with infectious Nipah viruses was performed in the Biosafety Level 4 (BSL4) laboratory at the Canadian Science Centre for Human and Animal Health, National Microbiology Laboratory of the Public Health Agency of Canada (PHAC). Animal protocol H-17-005 and procedures were approved by the institutional Animal Care Committee of the Canadian Science Centre for Human and Animal Health (CSCHAH), according to the guidelines of the Canadian Council on Animal Care.

### Cell lines, viruses, antibodies

Vero E6 (African green monkey kidney) cells were cultured in Dulbecco’s minimal essential medium (DMEM) (Sigma) supplemented with 10% fetal bovine serum (FBS) and 2 mM L-glutamine (Life Technologies). GripTite 293 (Human Embryonic Kidney) cells (293GT) (ThermoFisher) were cultured in DMEM supplemented with 10% FBS, 0.1 mM MEM Non-Essential Amino Acids (NEAA), and 600 μg/ml Geneticin (Life Technologies). BHK-21 (baby hamster kidney) cells were cultured in Dulbecco’s minimal essential medium (DMEM) (Sigma) supplemented with 10% fetal bovine serum (FBS) and 2 mM L-glutamine (Life Technologies). All cells were maintained at 37 °C and 5% CO_2_. The NiV-M isolate (NiV/MY/HU/1999/UMMC2; Accession #: AY029768) was generously provided by the US Center for Disease Control (CDC) and was passaged a total of 3 times in Vero E6 prior to being used in experiments. The mouse monoclonal antibodies against the nucleoprotein of NiV-M, F45G2 and fluorescein-conjugated F45G4, have been previously reported^54^. The mouse pan-actin monoclonal antibody was clone ACTN05 (Thermo Scientific).

### Bioinformatics analyses

Sequence alignments were performed using ClustalW within the MEGA Version 7.0 software package^68^ or with Clustal Omega version 1.2.4^69^. The phylogenetic tree was constructed based on full-length genomic sequences of the indicated NiV using the Neighbor-Joining method in MEGA version 7.0^68^ with 1000 bootstrap replicates. The tree is drawn to scale, with branch lengths corresponding to the evolutionary distances used to infer the phylogenetic tree. The evolutionary distances were calculated using the Maximum Composite Likelihood method^70^, and the units are the number of base substitutions per site. GenBank accession numbers for each sequence are listed below. Similarity plots of the indicated henipavirus genomes relative to NiV/BD/HY/2004/RA were generated using Simplot v 3.5.1^71^ using a window of 500 nt and a step of 50 nt after gap stripping.

### Construction of the NiV infectious clones

To generate the infectious NiV-M clone, four overlapping fragments of complementary DNA (cDNA), spanning the entire genome of minimally passaged NiV isolate, NiV/MY/HU/1999/C2 (accession no. AY029768), hereby referred to as fragments A-D (Fig. 1C), were amplified by reverse transcription PCR (RT-PCR) using PrimeSTAR Max polymerase (Takara). The resulting PCR products were gel purified with the QIAquick gel extraction kit (Qiagen) and purified fragments B and C were each cloned directly by In Fusion cloning (Clontech) into pSP-NA, a modified version of the high copy number pSP72 plasmid (Promega) that houses a minimal multiple cloning site (MSC) that includes NotI and AscI restriction sites and the eukaryotic RNA polymerase II cytomegalovirus immediate early promoter (polII). The pSP-NA plasmid containing genomic fragment A (3′ termini-SwaI) was generated by overlap extension PCR^72^ and In-Fusion HD cloning (Clontech) to include a self-cleaving hammerhead ribozyme (HHrbz) (Supplementary Fig. S1) immediately downstream of the transcription start site that ends at the insertion point of the 3′ end of genomic fragment A. The pSP-NA plasmid containing genomic fragment D (BsiWI to 5′ termini) was generated similarly to include a hepatitis delta virus ribozyme (HdRz) sequence (Supplementary Fig. S1) followed by a beta globin transcription terminator sequence immediately downstream of the 5′ end of genomic fragment D.

To generate the infectious NiV-B clone, a group of contiguous 40 mer oligonucleotides with 20 nt overlapping regions were synthesized to span the entire genome of NiV/BD/HY/2004/RA (accession no. AY988601) using gene2oligo^73^. The synthesized cDNA sequence was designed to match the viral genomic sequence retrieved from GenBank (accession no. AY988601) with the exception of two modifications introduced into the F protein by rolling circle mutagenesis, S207L and G252D, that corrected two amino acids sequence errors in the deposited GenBank sequence. The 40 mer oligonucleotides were then assembled into genomic fragments A-D with Taq Ligase (NEB) then amplified using iProof High-Fidelity DNA polymerase (Bio-Rad), according to the manufacturer’s instructions and cloned into pSP-NA, as described above.

The overlapping cDNA segments (A-D) spanning the entire genomes of NiV-M or NiV-B were sequentially assembled via In-Fusion cloning. All the infectious clone constructs were fully sequenced by Sanger sequencing with a set of primers that generated overlapping 700 nt amplicons that spanned the genome using a ABI 3130 Genetic Analyzer (Applied Biosystems).

Recombinant NiV-M and NiV-B cDNA constructs expressing enhanced green fluorescent protein (EGFP) were constructed, as above except an EGFP coding sequence was inserted by overlap extension PCR downstream of the G protein coding region, either in frame with G with a P2A cleavage site inserted between G and EGFP (pSPNiV-M/G-P2A-EGFP and pSPNiV-B/G-P2A-EGFP) or as a separate gene with the G-L intergenic sequence duplicated and placed between G and EGFP (pSPNiV-M/EGFP and pSPNiV-B/EGFP).

### Transfection of full-length infectious clones

To rescue infectious NiV (rgNiV-M, rgNiV-B, rgNiV-M/G-P2A-EGFP, rgNiV-B/G-P2A-EGFP, and rgNiV-M/EGFP) from the full-length cDNA clones, 2 × 10^5^ HEK 293 GripTite (293GT) cells (ThermoFisher) were seeded into one well of a 6-well plate 24 hours before transfection and maintained overnight at 37 °C with 5% CO_2_ in DMEM medium supplemented with 10% FBS, 0.1 mM MEM Non-Essential Amino Acids (NEAA), and 1x L-glutamine. On the following day, the medium was replaced with Opti-MEM (ThermoFisher), and the cells were co-transfected with NiV cDNA expression vector (pSP-NiV-M, pSP-NiV-B, pSPNiV-M/EGFP, pSPNiV-B/G-P2A-EGFP, or pSPNiV-M/G-P2A-EGFP) and the three helper plasmids, expressing the NiV-B N, P, or L proteins, pCAGGS-NiV-B-N, pCAGGS- NiV-B-P, pCAGGS- NiV-B-L, using 4 μg, 0.8 μg, 0.8 μg, and 0.4 μg, respectively, with TransIT LT1 transfection reagent (Mirus), as per the manufacturer’s instructions. At 1 day post-transfection DMEM supplemented with 1% FBS and 1x L-glutamine was added to the cells, and at 4 days post-transfection (d.p.t.) 1 × 10^5^ of fresh 293GT were added to the transfected cells. At 7 dpt. the cells were split at a 1:4 ratio into a larger flask. Finally, 7 days later (14 dpt), the supernatant was harvested and pelleted 1500 g for 5 minutes, and 500 μl of clarified supernatant was used to infect Vero E6 cells and cells were observed daily for cytopathic effect (CPE). The first passage (P1) virus was collected when CPE had reached approximately 90% and the supernatant was clarified by centrifugation, as above, and stored in aliquots at −80 °C. The virus was passaged once again on VeroE6 to generate the P2 stock virus. Virus titers were determined by TCID_50_ and plaque assay in VeroE6.

Alternatively, for the rescue of infectious rgNiV-B/EGFP and for the determination of rescue efficiency of rgNiV-M/EGFP and rgNiV-B/EGFP sub-confluent BHK-21 cells in a 6-well plate were co-transfected with NiV cDNA expression vector (pSPNiV-M/EGFP or pSPNiV-M/EGFP) and the three helper plasmids, expressing the NiV-B N, P, or L proteins, pCAGGS-NiV-B-N, pCAGGS- NiV-B-P, pCAGGS- NiV-B-L, using 3 μg, 1 μg, 0.2 μg, and 0.4 μg, respectively, with TransIT LT1 transfection reagent (Mirus) in 1 ml of Opti-MEM (ThermoFisher), as per the manufacturer’s instructions. The following day, 1 ml of MEM supplemented with 2% FBS, 0.1 mM MEM Non-Essential Amino Acids (NEAA), and 1x L-glutamine was added to each transfection well. From 4–7 dpi, 1 ml of supernatant was removed and replaced with MEM 2% FBS and was subsequently added to sub-confluent VeroE6 cells in DMEM supplemented with 2% FBS and 1x L-glutamine. Virus spread could be observed in BHK-21 cells via EGFP expression between 4–5 dpi, otherwise CPE and syncytia were not apparent. The first passage (P1) virus was collected when CPE in VeroE6 had reached approximately 90% and the supernatant was clarified by centrifugation, as above, and stored in aliquots at −80 °C. The virus was passaged once again on VeroE6 to generate the P2 stock virus.

### Virus titration by plaque assay, conventional TCID_50_ and EGFP-based TCID_50_

A plaque assay was performed to determine the titer of the rgNiV-M/EGFP and rgNiV-B/EGFP inoculums used in the PRNT assays. Briefly, ten-fold serial dilutions were made of rgNiV-M/EGFP or rgNiV-B/EGFP in DMEM, supplemented with 1x l-glutamine, 1% FBS, and 1x penicillin/streptomycin (p/s). One hundred microliter volumes of each dilution were then added in duplicate wells of a 12-well plate seeded with Vero E6 cells the day prior to be 95% confluent and incubated for 1 h at 37 °C with occasional gentle rocking. After one hour the inoculum was removed, and a 1 ml overlay with a working concentration of 1% SeaPlaque agarose (Lonza), 1x MEM, supplemented with 1x L-glutamine, 1% FBS, and 1x p/s was added to each well and the plates were incubated 37 °C with 5% CO_2_. A second overlay with 0.01% Neutral red stain was added at 2 dpi and the number of plaques were counted the following day.

For infectious virus assays, 50% tissue culture infective dose (TCID_50_) was calculated using the Reed and Muench method^74^. Briefly, cell culture supernatants or clarified tissue homogenates were serially diluted 10-fold in MEM supplemented with 1% heat-inactivated FBS, 1x L-glutamine and 1x p/s. One hundred microliter volumes of the dilutions were then added to each well of a 96-well plate seeded with Vero E6 cells the previous day to be 95% confluent in replicates of three and incubated for 1 h at 37 °C. The inoculum was then removed from each well, and fresh MEM supplemented with 1% FBS, 1x L-glutamine and 1x p/s was added and the cells were maintained at 37 °C with 5% CO_2_. At day 3–5 post-infection each well was scored for CPE (TCID_50_) or fluorescence (EGFP-based TCID_50_). The limit of detection for both TCID_50_ assays was 10^2.5^ TCID/ml.

### Virus replication kinetics

Vero E6 cells were seeded into 6-well plates to reach ~90% confluency the following day. After removing the medium, virus was added to the cells in triplicate wells and allowed to adsorb at 37 °C with periodic gentle rocking for 1 hour, followed by replacement of the medium with DMEM supplemented with 1% FBS and 1x L-glutamine. Samples of virus supernatant were collected every 24 hours for days 1–4, and fresh DMEM supplemented with 1% FBS and 1x L-glutamine was used to top up each well. Supernatants of rgNiV-M/EGFP or rgNiV-B/EGFP-infected cells were harvested for an additional 2 days. Virus titers in cell culture supernatants were determined by conventional TCID_50_ assay, as described above.

### Quantification and visualization of EGFP expression

Vero-E6 cells were seeded into 96-well plates to reach ~90% confluency the following day. After removing the medium, rgNiV-M/EGFP or rgNiV-B/EGFP was added to the cells in 6 wells (MOI = 0.01) in duplicate and allowed to adsorb at 37 °C with periodic gentle rocking for 1 hour, followed by replacement of the medium with DMEM supplemented with 1% FBS and 1x L-glutamine. The intensity of GFP fluorescence was measured using a Synergy HT microplate reader (BioTek) with a 485/20 excitation filter and a 516/20 emission filter, as per the manufacturer’s instructions.

Epifluorescence microscopy to visualize EGFP expression in infected cells was carried out on an EVOS FL imaging system using a GFP imaging cube (ThermoFisher Scientific).

### Serial passage of rgNiV-M/EGFP and rgNiV-B/EGFP

Vero E6 cells were seeded into 6-well plates then infected with rgNiV-M/EGFP, rgNiV-B/EGFP or mock-infected at an MOI of 0.01. At approximately 48- hpi, when advanced CPE was observed, cell supernatants were transferred to fresh Vero E6 cells. This process was repeated an additional 3 times, resulting in P6 rgNiV-M/EGFP and rgNiV-B/EGFP viruses. The EGFP fluorescence expression from P6 virus-infected cells was observed by fluorescence microscopy on an EVOS FL imaging system using a GFP imaging cube (ThermoFisher Scientific).

### Virus neutralization assays

Fifty plaque-forming units (PFU) of the recombinant EGFP-expressing NiV, rgNiV-M/EGFP or rgNiV-B/EGFP, was incubated with increasing dilutions of heat-inactivated serum from hamsters previously exposed to an attenuated strain of rgNiV-M or rgNiV-B in DMEM supplemented with 1% FBS for 1 hour at 37 °C. The virus-serum mixture was added to confluent Vero E6 cells in a 12-well plate in duplicate and incubated for 1 hour at 37 °C. The inoculum was removed, and a 1 ml overlay with a working concentration of 1% Seaplaque agarose (Lonza), 1x MEM, supplemented with 1x l-glutamine, 1% FBS, and 1x p/s was added to each well and the plates were incubated at  37 °C with 5% CO_2_. For the fluorescence focus reduction neutralization test (FFRNT) fluorescence foci were counted at 3 dpi, and for the conventional plaque reduction neutralization test (PRNT) assay plaques were counted at 5 dpi and the number of fluorescence foci or viral plaques were plotted as a percentage of the total number of PFU or fluorescence-forming units (FFU) counted in control wells, where virus had been incubated with non-neutralizing serum. A line of best fit was generated using GraphPad software.

### Hamster challenge studies

Five-to-six-week old female Syrian golden hamsters (*Mesocricetus auratus*) (Charles River) were challenged via the intranasal (i.n.) route with 10-fold serial dilutions (10^1^ to 10^5^ TCID_50_) of NiV-M (n = 3), rgNiV-M (n = 5 or 6), and rgNiV-B (n = 5 or 6) in a volume of 100 μl, with 50 μl delivered to each nare using a pipette. Post-challenge, animals were weighed daily and observed to assess any clinical signs of disease. Clinical signs were scored as follows: weight loss 0–9% (0), 10–14% (1), 15–20% (4), >20% (10); hunched posture (2); ruffled coat (2); aggression to handlers (2); reduced movement (2); laboured breathing (2); disorientation (2); wobbling gait (4); unresponsive (10); hind-limb paralysis (10); dyspnea (10); seizure (10); hemorrhaging (10). Euthanasia was carried out at a score of 10. The median (50%) lethal dose (LD_50_) was calculated by the Reed and Muench method^74^. Terminal blood samples were obtained from hamsters at 3 days post-infection by cardiac bleed under deep isoflurane anesthesia just prior to euthanasia. Necropsies were performed and tissues were harvested for downstream assays, as described below.

### Measurement of viral burden in tissues

For infectious assays portions of the brain, lung, liver, and spleen of infected hamsters were harvested at the time of necropsy and flash frozen and stored at −80 °C for later use. The tissue samples were later thawed and weighted and then disrupted in a TissueLyzer II homogenizer system (Qiagen) in 500 µl of DMEM supplemented with 1% heat-inactivated FBS, 1x L-glutamine, and 1x p/s with a stainless steel bead for 6 minutes at 30 cycles/s, as per the manufacturer’s instructions. Cell debris was pelleted by centrifugation in samples and standard TCID_50_ assays were performed with the clarified lysates, as described above.

### Western blotting

Whole-cell lysates were assayed for the NiV N protein by SDS-PAGE and Western blotting. At 24 or 48 hpi NiV-M isolate, rgNiV-M, or rgNiV-B-infected cells were washed with 1 x PBS and lysed in 4x SDS loading buffer (250 mM Tris-HCL (pH 7.5), 4% SDS, 35% glycerol, 0.5% Bromophenol blue). Samples were boiled for 10 minutes at 100 °C then transferred to clean tubes to be brought out of the BSL4 laboratory. Samples were boiled again at 100 °C for 10 minutes then and then 20% β-mercaptoethanol was added to each sample. The samples were resolved using SDS polyacrylamide gel electrophoresis (PAGE) on a 10% Bis-Tris NuPage gel (Invitrogen) using a Mini gel tank (Invitrogen) and transferred to a nitrocellulose (NC) membrane using an iBlot 2 transfer device (Invitrogen). The NC membrane was blocked with Odyssey Blocking Buffer (PBS) (LiCor) for 1 hour at RT. The NiV N protein was detected using monoclonal antibody against the NiV nucleoprotein (F45G2)^54^ at a 1:2000 dilution in Odyssey Blocking Buffer (PBS), and actin was detected using the Ab-5 pan-actin antibody (Thermo Fisher Scientific) at a 1:400 dilution Odyssey Blocking Buffer (PBS). All washes were performed with 0.1% Tween20 in PBS. Secondary antibody staining was done using IRdye 800CW goat anti-mouse (ex. 778, em. 806) (Mandel Scientific) followed by imaging on a LiCor Odyssey imager (LiCor Biosciences).

### Immunofluorescence analysis and fusion assays

Vero E6 cells were seeded into 96-well plates then mock-infected or infected with the various NiV at an MOI of 0.1. At 48 hpi, cells were fixed in 10% phosphate-buffered formalin solution for greater than 24 hours, followed by buffer change with fresh 10% phosphate-buffered formalin to inactivate virus for removal from the BSL4 laboratory. The 96-well plates were stored for an additional 24 hours in the formalin solution at 4 °C after which the cells were washed four times with phosphate-buffered saline (PBS), then the cell membranes were permeabilized with 0.1% Triton X-100 for 10 minutes at room temperature, washed three additional times with PBS then stained with monoclonal fluorescein-conjugated anti-NiV-N antibody F45G4 (1:100) for 2 hours at room temperature. Cells were then stained with a 300 nM solution of 4’,6-diamidino-2-phenylindole, dihydrochloride (DAPI) (ThermoFisher Scientific) diluted in PBS, washed three times with PBS, and images were acquired using a Zeiss LSM 700 confocal microscope.

For fusion assays, immunofluorescence analysis was performed as above except cells were co-stained with phalloidin-594 to allow for demarcation of individual cells by staining of f-actin. Five random microscopic fields were randomly selected at 40x magnification, and the number of nuclei within each field that were contained cells that stained positive for NiV N antigen and had greater than three nuclei were tallied, and the average of the four microscopic fields were graphed.

### Histology and viral RNA *in situ* hybridization

Tissues were fixed in 10% neutral phosphate buffered formalin for 30 days, routinely processed, sectioned at 5 μm, then stained with hematoxylin and eosin for histopathologic examination or manipulated as described below for RNA *in situ* hybridization (RNA ISH). RNA ISH was carried out using RNAscope 2.5 HD Detection Reagent-Red (Advanced Cell Diagnostics), according to the manufacturer’s instructions. Briefly, formalin-fixed, paraffin-embedded tissue samples of brain and lung mounted on slides were baked in a dry oven for 1 hour at 60 °C and were deparaffinized. Tissue sections were pre-treated with RNAscope H_2_O_2_ to block endogenous peroxidases for 10 minutes at room temperature. Target retrieval was carried out using the RNAscope Target Retrieval Reagent for 15 minutes. RNAscope Protease Plus Reagent was then applied for 30 minutes at 40 °C. The probe targeting NiV RNA was designed and manufactured by Advanced Cell Diagnostics (Reference # 439251). The negative probe was also obtained from Advanced Cell Diagnostics (Reference # 310034). Tissues were counterstained with Gills I Hematoxylin and images were captured using a light microscope.

### Data analysis

Data were analyzed and graphed using Prism 7 software (GraphPad Software). Statistical analyses were performed, as appropriate, using two-way analysis of variance (ANOVA) with Bonferroni’s posttest, one-way ANOVA with Dunnett’s test, or the Mann-Whitney test.

### Accession codes

The GenBank Accession numbers are as follows: NV/MY/PI/99/UM-0128 (AJ564623), NV/MY/PI/99/VRI-2794 (AJ564621), NiV/MY/HU/99/C1 (AY029767), NiV/MY/HU/1999/C2 (AY029768), NiV/MY/PI/99/VRI-1413 (AJ564622), NiV/BD/HU/2004/RA (AY988601), NiV/IN/HU/2007/FJ (FJ513078), NiV/BD/HU/2008/Manikgonj (JN808857), NiV/BD/HU/2008/Rajbari (JN808863), and HeV/AU/HO/1997 (AF017149.3).

## Supplementary information

Supplementary Figure S1

## Data Availability

All relevant data are available from the authors upon request.
