# Supplementary material for: Establishment of an RNA polymerase II-driven reverse genetics system for Nipah virus strains from Malaysia and Bangladesh
Source: Sci Rep. 2019 Aug 1;9:11171. doi: 10.1038/s41598-019-47549-y (PMC6671980; doi:10.1038/s41598-019-47549-y)
Supplement: Supplementary file 1 — Supplementary Figure S1 [file 41598_2019_47549_MOESM1_ESM.pdf]

Establishment of an RNA polymerase II-driven reverse genetics system for Nipah virus strains from Malaysia and Bangladesh

Bryan D. Griffin[1,2,+,] Anders Leung[1+], Mable Chan[1, 2], Bryce M. Warner[1,2], Charlene Ranadheera[1], Kevin Tierney[1], Jonathan Audet[1, 2], Kathy L. Frost[3], David Safronetz[1,2], Carissa Embury-Hyatt[4], Stephanie A. Booth[2,3], Darwyn Kobasa[1,2, *]

[1]Zoonotic Diseases and Special Pathogens Program, National Microbiology Laboratory, Public Health Agency of Canada, 1015 Arlington Street, Winnipeg, Manitoba, Canada R3E 3R2.

[2]Department of Medical Microbiology and Infectious Diseases, University of Manitoba, 745 Bannatyne Avenue, Winnipeg, Manitoba, Canada R3E 0J9

[3]Molecular Pathobiology, National Microbiology Laboratory, Public Health Agency of Canada, Canada, 1015 Arlington Street, Winnipeg, Manitoba, Canada R3E 3R2

[4] National Centre for Foreign Animal Disease, Canadian Food Inspection Agency, 1015 Arlington Street, Winnipeg, Manitoba, Canada R3E 3M4

*Corresponding author: darwyn.kobasa@canada.ca

+these authors contributed equally to this work

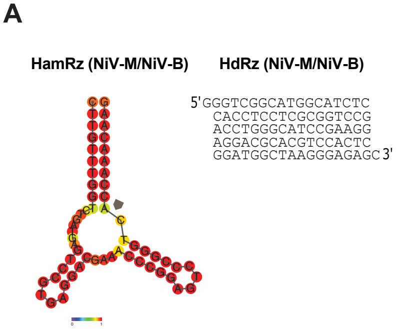

B

	Rescue efficiency (pos/total)	Time to rescue (days)
rgNiV-M/EGFP	3/4	5-7
rgNiV-B/EGFP	3/4	5-7

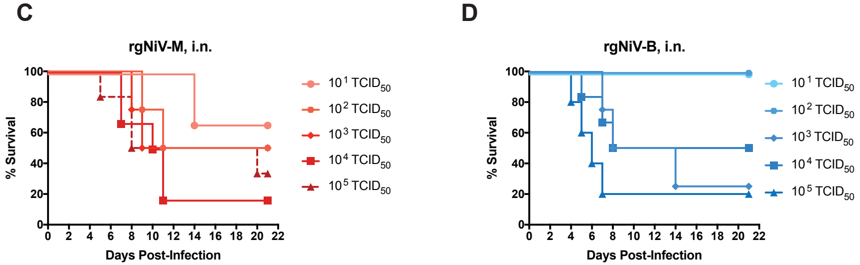

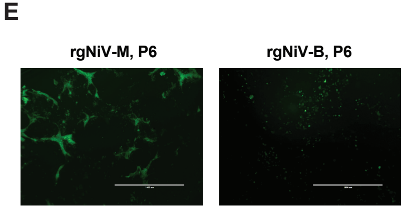

Supplementary Figure S1. **A**, Complete sequence of the hammerhead ribozyme (HamRz) (left panel) and the hepatitis delta virus ribozyme (HdRz) (right panel) sequences used to generate the rgNiV constructs. The HamRz structure was predicted using RNAFold 2.4.11. The cleavage site is indicated by a grey arrowhead. **B**, Efficiency of rescue. Rescue efficiency is depicted as the number of positive wells (pos) divided by the number of total wells. The time to rescue in BHK cells is indicated. **C/D**, Kaplan-Meier curves representing survival data are shown. Groups of five-to-six-week week-old female hamsters were inoculated with the indicated doses of rgNiV-M (C, n= 5-6) or rgNiV-B (D, n= 5-6) by the intranasal route (i.n.). Hamsters were observed daily to assess the clinical signs and survival. **E**, Stability of EGFP-expressing viruses. The rgNiV-M/EGFP or rgNiV-B/EGFP were serially passaged in Vero E6 and the EGFP fluoresence of the passage 6 (P6) viruses was visualized by fluorescence microscopy.
